# Enhancing the direct charging performance of an open quantum battery by adjusting its velocity

**DOI:** 10.1038/s41598-023-47193-7

**Published:** 2023-11-14

**Authors:** B. Mojaveri, R. Jafarzadeh Bahrbeig, M. A. Fasihi, S. Babanzadeh

**Affiliations:** Department of Physics, Azarbaijan Shahid Madani University, PO Box 51745-406, Tabriz, Iran

**Keywords:** Information theory and computation, Quantum physics, Optics and photonics

## Abstract

The performance of open quantum batteries (QBs) is severely limited by decoherence due to the interaction with the surrounding environment. So, protecting the charging processes against decoherence is of great importance for realizing QBs. In this work we address this issue by developing a charging process of a qubit-based open QB composed of a qubit-battery and a qubit-charger, where each qubit moves inside an independent cavity reservoir. Our results show that, in both the Markovian and non-Markovian dynamics, the charging characteristics, including the charging energy, efficiency and ergotropy, regularly increase with increasing the speed of charger and battery qubits. Interestingly, when the charger and battery move with higher velocities, the initial energy of the charger is completely transferred to the battery in the Markovian dynamics. In this situation, it is possible to extract the total stored energy as work for a long time. Our findings show that open moving-qubit systems are robust and reliable QBs, thus making them a promising candidate for experimental implementations.

## Introduction

In recent years, with advancements in quantum thermodynamics, there has been a radical change of perspective in the framework of energy manipulation based on the electrochemical principles. The possibility to create an alternative and efficient energy storage device at small scale introduces the concept of the quantum battery (QB), which was proposed by Alicki and Fennes in the 2013’s^1^, and subsequently became into a significant field of research. As their name indicates, QBs are finite dimensional quantum systems that are able to temporarily store energy in their quantum degrees of freedom for later use. The fundamental strategy for developing the idea of QBs is based on their non-classical features such as quantum coherence, entanglement and many-body collective behaviors that can be cleverly exploited to achieve more efficient and faster charging processes than the macroscopic counterparts^2–7^. A QB is charged based on an interaction protocol between QB itself with either an external field or a quantum system which serves as a charger. It is then discharged into a consumption hub based on the same protocol. When the battery enters into an interaction with the charger, it transits from a lower energy level into the higher ones and will be charged. So far, a variety of powerful charging protocols have been proposed in different platforms, including two-level systems^8–10^, harmonic oscillators^11^, and hybrid light-matter systems^12–15^. Some efforts have been also devoted to implement QBs, based on different quantum systems, for example, using the optical and solid-state systems, such as quantum electrodynamics (QED) setups^16–19^, NMR spin systems^20^ and superconducting devices^21–23^.

Due to the fact that a real quantum system inevitably interacts with its environment, studying QBs from the open quantum systems perspective is attracting considerable interest. The interaction of a QB with its surrounding environments causes the leakage of the coherence of battery to the environment, leading to decoherence effect in the battery. Such an adverse effect often plays a negative role in the charging and discharging performance of QBs^24–26^. Decoherence brought during the charging process tends to lead QBs to a non-active (passive) equilibrium state in which work extracting from the QBs is often impossible^27^ in a cyclic unitary process. The environmental-induced noises also affect QBs that are disconnected from both charger and consumption hub and cause self-discharging of that QBs^28–30^. Therefore, designing a more robust battery against the environmental dissipations is valuable step for implementation of QBs in the real-life. Recently, researchers have devoted efforts not only to studying the effect of the environment on QBs, but also to exploiting non-classical effect as well as to developing open system protocols to stabilize the charging cycle performance through quantum control techniques. For example, Kamin et al.^31^ studied the charging performance of a qubit-based QB charged by the mediation of a non-Markovian environment. They revealed the non-Markovian property is beneficial for improving charging cycle performance. In Ref.^32^, the authors studied dynamics of a continuous variable QB coupled weakly to the squeezed thermal reservoir and managed to control the performance of the charging process by boosting the quantum squeezing of reservoir. A feasible route for harnessing loss-free dark states for stabilizing the stored energy of a qubit-based open QB has been introduced in^33^. In addition to the above studies, several other protocols have been developed to protect the charging cycle of QBs such as feedback control method^34–36^, convergent iterative algorithm^37^, Bang–Bang modulation of the intensity of an external Hamiltonian^38^, inhiring an auxiliary quantum system^39^, modulating the detuning between system and reservoir^40^, stimulated Raman adiabatic passage technique^41^, engineering quantum environments^42^, etc.

On the other hand, according to the previous studies on the Markovian and non-Markovian dynamics of open two-qubit systems, translational motion of qubits provides novel insights for stabilizing entanglement and coherence of a two-qubit system against the environmental induced dissipations by suitably adjusting the velocities of the qubits^43–52^. We want here to use this safeguard capability of the motional properties to improve the charging cycle performance of the open qubit-based QBs. Recently, the effect of translational motion of qubits on the performance of qubit-based open QB has been examined in^53^, where the charger and battery’s qubits move with a particular speed inside a common leaky cavity. In this model, the battery and charger have no direct interaction with each other, and the battery is charged via the environment-mediated charging process. The authors have found that the movement of the quantum battery inside the cavity has a negative effect on the performance of the quantum battery during the charging process. In the present work, we consider a moving-biparticle system composed of a qubit-battery and a qubit-charger that independently interact with their local environments. The battery qubit is charged assisted by the dipole–dipole interaction with the charger qubit. We will investigate how the translational motion of qubits affects the charging process of QB. Our results show that translational motion of qubits always plays a constructive role in protecting QB from decay induced by the environment. This work is organized as follows: in Section “Figures of merit”, we introduce and describe several figures of merit for characterizing the performance of QBs. In Section “Open moving-quantum battery”, we illustrate our model and obtain explicit expressions for the reduced density matrix of the QB and the charger. In Section “Numerical results and discussion” we present the results of our numerical simulations in the context of their physical significance. Finally, Section “Outlook and summary” concludes this paper.

## Figures of merit

Let us consider a QB modeled as a quantum system with d-dimensional Hilbert space $\mathcal {H}$ and Hamiltonian $H_B$ such that

1 $$\begin{aligned} H_B=\sum _{i=1}^{d} \varepsilon _i |\varepsilon _i\rangle \langle \varepsilon _i|, \end{aligned}$$

with non-degenerate energy levels $\varepsilon _i \le \varepsilon _{i+1}$. Internal energy of QB is given by $Tr(\rho _B H_B)$, where $\rho _B$ is the state of the battery. Charging a QB means bringing the quantum system from a lower energy state $\rho _B$ to a higher energy state $\rho _B^\prime$, while discharging refers to the inverse process, i.e., brings the quantum system from a higher energy state $\rho _B^\prime$ to a lower one $\rho _B^{\prime \prime }$:

2 $$\begin{aligned} \texttt {Tr}\left\{ \left( \rho _B^\prime -\rho _B\right) H_B\right\} \ge 0,\qquad \qquad \qquad \qquad charging\,\, process\nonumber \\ \texttt {Tr}\left\{ \left( \rho _B^{\prime }-\rho _B^{\prime \prime }\right) H_B\right\} \ge 0.\qquad \qquad \qquad \quad \;\;discharging\,\, process \end{aligned}$$

Therefore, in a charging process, the actual stored energy of QB at time *t*, regarding the initial energy, can be expressed as follows^1^

3 $$\begin{aligned} \Delta E_B=\texttt {Tr}\{\rho _B(t) H_B\}-\texttt {Tr}\{\rho _B(0) H_B\}. \end{aligned}$$

According to the second law of thermodynamics, a complete converting of the stored energy into the valuable work without dissipation of heat is impossible. The maximum amount of energy extracted from a given quantum state $\rho _B=\sum _{i} r_i |r_i\rangle \langle r_i|$, ($r_i \ge r_{i+1}$) through a cyclic unitary operation is called ergotropy^54^. This quantity can be defined as^54–56^

4 $$\begin{aligned} \mathcal {W}=\texttt {Tr}\{\rho _B H_B\}-\texttt {min}_U\,\texttt {Tr}\{U\rho _B U^{\dagger } H_B\}, \end{aligned}$$

where the minimization is taken over all possible unitary transformations acting locally on such system. It has been shown in^54^ that no work can be extracted from the passive counterpart of $\rho _B$ with the form $\sigma _{\rho _B}=\sum _{i} r_i |\varepsilon _i\rangle \langle \varepsilon _i|$. The unique unitary transformation $U=\sum _i |\varepsilon _i\rangle \langle r_i|$ on the $\rho$ minimizes $\texttt {Tr}(U\rho _B U^{\dagger } H_B)$, and when inserted in Eq. (4) yields the following expression for the ergotropy

5 $$\begin{aligned} \mathcal {W}=\sum _{i,j} r_j \varepsilon _i\left( |\langle r_j|\varepsilon _i\rangle |^2-\delta _{ij}\right) . \end{aligned}$$

In order to quantify the amount of extractable energy, the efficiency $\eta$ is defined as the ratio between the ergotropy $\mathcal {W}$ and the total charging energy $\Delta E_B$

6 $$\begin{aligned} \eta =\frac{\mathcal {W}}{\Delta E_B}. \end{aligned}$$

It is worth mentioning that this definition of efficiency makes sense for the QBs prepared initially in a passive state, since it is the fraction of the energy stored in the QB that later is converted to the ergotropy. When a QB is initiated in an active state (such as coherent state) the ergotropy $\mathcal {W}$ may be larger than $\Delta E_B$, and the efficiency becomes beyond one. In this situation, one can use $\eta =\frac{\mathcal {W}}{E_B}\le 1$ to quantify efficiency^28^. In the case $\eta =1$ we have $\mathcal {W}=E_B$, whereas $\eta <1$ indicates that there is an amount of “dead energy” that can not be later extracted by unitary operations.

**Figure 1:**
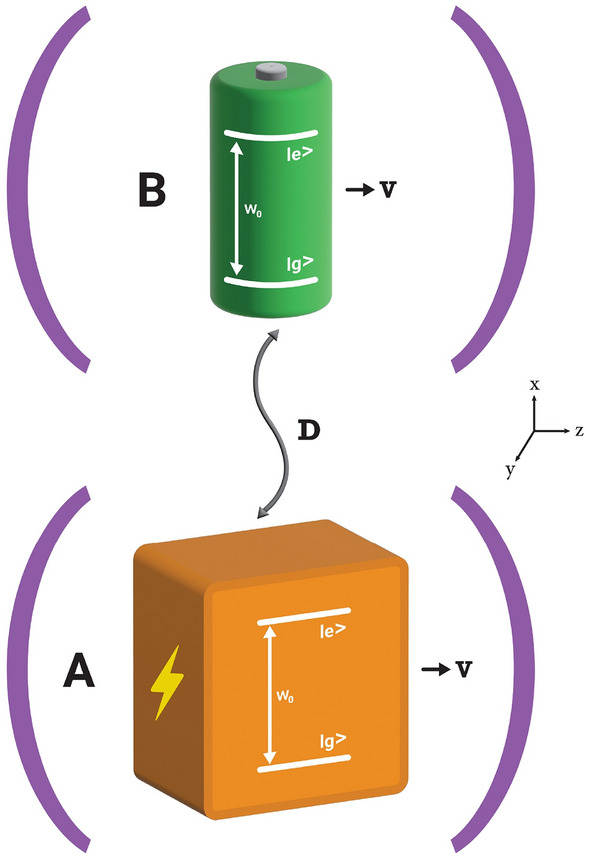
Schematic illustration of a qubit-based open QB composed of a qubit-battery and a qubit-charger moving along the z-axis of two distinct but identical cavity reservoirs. The qubits move with constant speed *v* and are also coupled to each other through the dipole–dipole interaction.

## Open moving-quantum battery

The open QB under consideration is composed of an atomic two-qubit system, the qubit *A* as a charger and the qubit *B* as a quantum battery, coupled to each other through the dipole–dipole interaction. The battery and charger qubits are coupled locally to two independent zero-temperature cavity reservoirs (see Fig. 1). We assume that each qubit moves along the *z*-axis of its cavity at a constant non-relativistic speed *v*. For simplicity we neglect here any scattering^57^ or trapping^58^ effects and consider the translational motion of the atomic qubits being classically. Turning on the dipole–dipole coupling between the charger and battery initiates the charging process.

Under the dipole and rotating wave approximation, the entire system is ruled by Hamiltonian (setting $\hbar =1$)

7 $$\begin{aligned} H=H_0+H_{int}, \end{aligned}$$

with

8 $$\begin{aligned}{} & {} H_0=H_A+H_B+H_{R_A}+H_{R_B}=\sum _{j=A,B}\left( \frac{\omega _0}{2} \sigma _{z}^{j} + \sum _{k}\omega _{k}^j a_{k}^{j\dag } a_{k}^j\right) ,\nonumber \\{} & {} H_{int}=H_{A-B}+H_{A-R_A}+H_{B-R_B}=D\left( \sigma _{+}^{A}\sigma _{-}^{B}+\sigma _{-}^{A} \sigma _{+}^{B}\right) +\sum _{j=A,B}\sum _{k} f_k^j(z) \left( \mathfrak {g}_{k}^j \sigma _{+}^{j} a^j_k +H.c.\right) . \end{aligned}$$

Here, H.c. stands for Hermitian conjugate, $\sigma _z^j$, $\sigma _+^j$, and $\sigma _-^j$ $(j=A,B)$ are, respectively, the population inversion, raising and lowering operators of the *j*th qubit with transition frequency $\omega _0$. $a_k^{j\dagger }$ and $a^j_k$ are, respectively, the creation and annihilation operators of the *k*th mode of the cavity reservoir *j* with the frequency $\omega _k^j$. Also, *D* is coupling constant of the dipole–dipole interaction between the battery and charger qubits, and $\mathfrak {g}_{k}^j$ is the coupling constant between the *j*th qubit and *k*th mode of the cavity reservoir *j*. The effect of translational motion of the battery and charger qubits has been included in the model by introducing the *z*-dependent shape function $f_k^j(z)$ in the Hamiltonian $H_{int}$. When the battery and charger qubits are moving with a same constant velocity *v*, the shape function $f_k^j(z=vt)$ can be taken into account as

9 $$\begin{aligned} f_k^j(z)=\sin [\omega _k^j(\beta t-\Gamma )],\qquad \qquad j=A,B \end{aligned}$$

where, $\Gamma =L/c$ with *L* being the size of the cavity. Also, $\beta =v/c$ where *c* refers to the speed of light in the vacuum space. This particular form of the shape function can be obtained by imposing an appropriate boundary condition on the cavity reservoirs^45,59^. Here we describe the translational motion of both battery and charger qubits by classical mechanics ($z=vt$). To this end, we will choose the values of the parameters in such a way that the de Broglie wavelength of qubit $\lambda _B$ is significantly smaller than the wavelength $\lambda _0$ associated with the resonant transition $\omega _0=\omega _n$ ($\omega _n$ is the central frequency of the cavity field mode)^60,61^. Furthermore, we consider a situation in which the photon momentum is relatively smaller than the atomic momentum and thus we neglect the atomic recoil caused by the interaction with the electric field^62^. In the optical regime, to ignore the atomic recoil and consider the translational motion of atoms classically, the velocity of qubits should be $v\gg 10^{-3}$^45^.

In the interaction picture (IP) generated by the unitary transformation $U=e^{-iH_0t}$, the Hamiltonian (8) can be written as follows

10 $$\begin{aligned}{} & {} \hspace{-1.5cm}H_{IP}=D\left( \sigma _{+}^{A} \sigma _{-}^{B}+\sigma _{-}^{A} \sigma _{+}^{B}\right) + \sum _{j=A,B}\sum _{k} f_k^j(z)\left( \mathfrak {g}_{k}^j \sigma _{+}^{j} a_k^{j} e^{i(\omega _0-\omega _k^j)t}+\mathfrak {g}_k^{j *} \sigma _{-}^{j}a_{k}^{j\dag } e^{-i(\omega _0-\omega _k^j)t}\right) . \end{aligned}$$

It is straightforward to show that the total excitation operator $\mathcal {N}=\sum _{j=A,B}\left( \sum _ka_k^{j\dagger }a_k^j+ \frac{1}{2}\sigma_{z}^{j}\right) +1$, commutes with the total Hamiltonian, i.e. $[H,\mathcal {N}]=0$ and therefor it is the constant of the motion. This allows us to decompose Hilbert space of the entire qubit-cavity system, $\mathcal {H}=\mathcal {H}_q\otimes \mathcal {H}_R$ spanned by the basis $\{\left| i_A,j_B\right\rangle \otimes \left| n_1,n_2, \ldots ,n_k, ...\right\rangle _{R_A}|_{n_1,n_2, \ldots =0}^{\infty } \otimes \left| m_1,m_2, \ldots ,m_k, ...\right\rangle _{R_B}|_{m_1,m_2, \ldots =0}^{\infty }\}$ $\left( i,j=e,g\right)$ into the excitation subspaces, as follows

11 $$\begin{aligned}{} & {} \hspace{-14mm} \mathcal {H}=\oplus _{n=0}^{\infty } \mathcal {H}_{n}. \end{aligned}$$

As a result of this decomposition, the dynamics of the entire qubit-reservoir system can be restricted to the excitation subspaces labeled by the total excitation number *n*. Here we are interested to explore dynamics of the entire system in the single-excitation subspace $\mathcal {H}_1$ spanned by vectors $\{\left| g_A,g_B\right\rangle \otimes \left| 1_k\right\rangle _{R_A}\left| 0_k\right\rangle _{R_B}|_{k=0}^\infty , \left| g_A,g_B\right\rangle \otimes \left| 0_k\right\rangle _{R_A}\left| 1_k\right\rangle _{R_B}|_{k=0}^\infty , \left| e_A,g_B\right\rangle \otimes \left| 0_k\right\rangle _{R_A}\left| 0_k\right\rangle _{R_B}, \left| g_A,e_B\right\rangle \otimes \left| 0_k\right\rangle _{R_A}\left| 0_k\right\rangle _{R_B}\}$ in which the single excitation is either in one of the qubits or in the k-th mode of one of cavity reservoirs. We consider a normalized initial state of entire qubit-reservoir as a superposition of $\left| e_A,g_B\right\rangle \otimes \left| 0_k\right\rangle _{R_A}\left| 0_k\right\rangle _{R_B}$ and $\left| g_A,e_B\right\rangle \otimes \left| 0_k\right\rangle _{R_A}\left| 0_k\right\rangle _{R_B}$ states with the following form

12 $$\begin{aligned} |\Psi (0)\rangle =\big [c_1(0) |e_{A},g_{B}\rangle +c_2(0) |g_{A},e_{B}\rangle \big ]\otimes |0\rangle _{R_A}|0\rangle _{R_B}. \end{aligned}$$

For times $t>0$, we expand the state vector $|\Psi (t)\rangle$ in terms of the vector basis of the single-excitation subspace $\mathcal {H}_1$ as

13 $$\begin{aligned}{} & {} \hspace{-3.5cm}\left| \Psi (t)\right\rangle =\big [c_1(t)\left| e_{A}, g_B\right\rangle +c_2(t) \left| g_A, e_B\right\rangle \big ] \otimes \left| 0_k\right\rangle _{R_A}\left| 0_k\right\rangle _{R_B} \nonumber \\{} & {} \hspace{-2.35cm}+\left| g_A, g_B\right\rangle \otimes \sum _{k} \big [d_{k}(t)\left| 1_k\right\rangle _{R_A}\left| 0_k\right\rangle _{R_B}+d_{k}^{\prime }(t) \left| 0_k\right\rangle _{R_A}\left| 1_k\right\rangle _{R_B}\big ], \end{aligned}$$

where the time-dependent amplitudes satisfy the normalization requirement

14 $$\begin{aligned} \sum _{i=1}^2|c_i(t)|^2+\sum _k(|d_{k}(t)|^2+|d_{k}^{\prime }(t)|^2)=1. \end{aligned}$$

By taking the partial traces over the field modes and subsystem A (B), the reduced time-dependent density operator for the charger (battery) in the $\{\left| e\right\rangle , \left| g\right\rangle \}$ basis is obtained as

15a $$\begin{aligned}{} & {} \hspace{-2cm}\rho _A(t)=|c_1(t)|^2\left| e_A\right\rangle \left\langle e_A\right| +\left( 1-|c_1(t)|^2\right) \left| g_A\right\rangle \left\langle g_A\right| ,\end{aligned}$$

15b $$\begin{aligned}{} & {} \hspace{-2cm}\rho _B(t)=|c_2(t)|^2\left| e_B\right\rangle \left\langle e_B\right| +\left( 1-|c_2(t)|^2\right) \left| g_B\right\rangle \left\langle g_B\right| . \end{aligned}$$

Inserting Eq. (13) into the time dependent Schrödinger equation $H_{IP}|\Psi (t)\rangle =i\frac{d}{d t}|\Psi (t)\rangle$, with $H_{IP}$ given in (10), leads to the following set of differential equations for time-dependent amplitudes

16a $$\begin{aligned}{} & {} \hspace{-4cm}i\dot{c_1}(t)=D c_2(t)+\sum _{k} \mathfrak {g}_{k}^A f_k^A(z)d_{k}(t)e^{i(\omega _0-\omega _{k}^A)},\end{aligned}$$

16b $$\begin{aligned}{} & {} \hspace{-4cm}i\dot{c_2}(t)= D c_1(t)+ \sum _{k} \mathfrak {g}_{k}^B f_k^B(z)d_{k}^{\prime }(t)e^{i(\omega _0-\omega _{k}^B)},\end{aligned}$$

16c $$\begin{aligned}{} & {} \hspace{-4cm}i\dot{d}_{k}(t)=\mathfrak {g}_k^{A*}f_k^A(z) c_1(t)e^{-i(\omega _0-\omega _{k}^A)t},\end{aligned}$$

16d $$\begin{aligned}{} & {} \hspace{-4cm}i\dot{d}_{k}^{\prime }(t)= \mathfrak {g}_k^{B*}f_k^B(z) c_2(t)e^{-i(\omega _0-\omega _{k}^B)t}. \end{aligned}$$

By integrating Eqs. (16c) and (16d) with the initial condition $d_{k}(0)=0$ and $d_{k}^{\prime }(0)=0$ and putting their solutions, respectively, in Eqs. (16a) and (16b), we get the following integro-differential equations for the amplitudes $c_1(t)$ and $c_2(t)$

17a $$\begin{aligned}{} & {} \hspace{-2cm}\dot{c_1}(t)=-iDc_2(t)-\int _{0}^{t}F_A(t-t^\prime )c_1(t^\prime )dt^\prime ,\end{aligned}$$

17b $$\begin{aligned}{} & {} \hspace{-2cm}\dot{c_2}(t)=-iDc_1(t)-\int _{0}^{t}F_B(t-t^\prime )c_2(t^\prime )dt^\prime , \end{aligned}$$

where

18a $$\begin{aligned}{} & {} \hspace{-2cm}F_{A}(t-t^\prime )=\sum _{k} |\mathfrak {g}_{k}^A|^2 e^{i(\omega _0-\omega _{k}^A)(t-t^\prime )}\sin [\omega _k^A(\beta ^A t-\Gamma )]\sin [\omega _k^A(\beta ^A t^\prime -\Gamma )],\end{aligned}$$

18b $$\begin{aligned}{} & {} \hspace{-2cm}F_{B}(t-t^\prime )=\sum _{k} |\mathfrak {g}_{k}^B| ^2 e^{i(\omega _0-\omega _{k}^B)(t-t^\prime )}\sin [\omega _k^B(\beta ^B t-\Gamma )]\sin [\omega _k^B(\beta ^B t^\prime -\Gamma )], \end{aligned}$$

are the memory correlation function of the reservoirs *A* and *B*, respectively. For simplicity, we suppose $F_{A}(t-t^\prime )=F_{B}(t-t^\prime )=F(t-t^\prime )$. In the limit of a large number of modes ( in the continuum limit ), the correlation function $F(t-t^\prime )$ takes the following form

19 $$\begin{aligned} F(t-t^\prime )=\int d\omega J(\omega ) e^{i(\omega _0-\omega )(t-t^\prime )}\sin [\omega (\beta t-\Gamma )]\sin [\omega (\beta t^\prime -\Gamma )], \end{aligned}$$

in which $J(\omega )$ is the spectral density of the cavity reservoirs and has the Lorentzian form^59,63^

20 $$\begin{aligned} J(\omega )=\frac{1}{2\pi }\frac{\gamma \lambda ^2}{(\omega _0-\omega -\Delta )^2+\lambda ^2}, \end{aligned}$$

where $\lambda$ defines the spectral width of the coupling which is connected to the memory time $\tau _E$ by the relation $\tau _E=\lambda ^{-1}$ and $\gamma$ refers to the qubit-environment coupling strength which is related to the relaxation time scale $\tau _R$ by $\tau _R \approx \gamma ^{-1}$. Also $\Delta$ is the detuning of $\omega _0$ and the central frequency of the cavity. The Markovian and non-Markovian dynamics of battery-charger system can be distinguished by comparing $\gamma$ and $\lambda$. When the coupling between qubits and reservoir is weak, i.e., $\frac{\gamma }{\lambda }\ll 1$, dynamics of the system is Markovian, where information or energy exponentially decays to the zero. However in the strong coupling regime, i.e., $\frac{\gamma }{\lambda }\gg 1$, dynamics of the system is non-Markovian. In this regime, the information or energy flows back from the environment to the system^63^.

By inserting the Eq. (20) into the Eq. (19) and after some calculations, in the continuum limit ($\Gamma \rightarrow \infty$), the correlation function is simplified as

21 $$\begin{aligned} F(t-t^\prime )=\frac{\gamma \lambda }{4} \cosh [\beta (\overline{\lambda }+i\omega _0)(t-t^\prime )] e^{-\overline{\lambda } |t-t^\prime |}, \end{aligned}$$

with $$\overline{\lambda }=\lambda -i\Delta$$.

In view of (21), taking the Laplace transformations of both sides of the differential Eqs. (17a) and (17b) and using the convolution property $\mathcal {L}[\int _{0}^{t}\textbf{A}(t-t^\prime ) \textbf{B}(t^\prime ) dt^\prime ]=\textbf{A}(s)\textbf{B}(s)$ yields

22a $$\begin{aligned}{} & {} \hspace{-2cm}sc_1(s)-c_1(0)=-iDc_2(s)-F(s)c_1(s),\end{aligned}$$

22b $$\begin{aligned}{} & {} \hspace{-2cm}sc_2(s)-c_2(0)=-iDc_1(s)-F(s)c_2(s), \end{aligned}$$

where the functions $c_1(s)$ and $c_2(s)$ are the Laplace transformations of the $c_1(t)$ and $c_2(t)$, respectively, and *F*(*s*) is the Laplace transforms of $F(t-t^\prime )$ which has the following explicit form

23 $$\begin{aligned} F(s)=\frac{\gamma \lambda }{4}\frac{s+\overline{\lambda }}{(s+\overline{\lambda })^2 -\beta ^2(\overline{\lambda }+i\omega _0)^2}. \end{aligned}$$

By reformulating the Eqs. (22a) and (22b), we get a general solution for $c_1(s)$ and $c_2(s)$ as follows

24a $$\begin{aligned}{} & {} \hspace{-2cm}c_1(s)=\frac{s+F(s)}{\big (s+F(s)\big )^2+D^2}c_1(0)-i\frac{D}{(s+F(s))^2+D^2}c_2(0),\end{aligned}$$

24b $$\begin{aligned}{} & {} \hspace{-2cm}c_2(s)=\frac{s+F(s)}{\big (s+F(s)\big )^2+D^2}c_2(0)-i\frac{D}{(s+F(s))^2+D^2}c_1(0). \end{aligned}$$

Then, by using the partial decomposition method, the Eqs. (24a) and (24b) can be decomposed into

25a $$\begin{aligned}{} & {} \hspace{-2cm}c_1(s)=\frac{1}{2}\bigg (\frac{1}{s+F(s)+iD}+\frac{1}{s+F(s)-iD}\bigg )c_1(0) +\frac{1}{2}\bigg (\frac{1}{s+F(s)+iD}-\frac{1}{s+F(s)-iD}\bigg )c_2(0),\end{aligned}$$

25b $$\begin{aligned}{} & {} \hspace{-2cm}c_2(s)=\frac{1}{2}\bigg (\frac{1}{s+F(s)+iD}+\frac{1}{s+F(s)-iD}\bigg )c_2(0) +\frac{1}{2}\bigg (\frac{1}{s+F(s)+iD}-\frac{1}{s+F(s)-iD}\bigg )c_1(0). \end{aligned}$$

In continuation, by applying the inverse Laplace transformation on the both side of the above equations, we obtain finally $c_1(t)$ and $c_2(t)$, as

26a $$\begin{aligned}{} & {} \hspace{-2cm}c_1(t)=\frac{1}{2}\bigg (\mathcal {M} _{+}(t)+\mathcal {M} _{-}(t)\bigg )c_1(0)+\frac{1}{2}\bigg (\mathcal {M}_+(t)-\mathcal {M}_-(t)\bigg )c_2(0), \end{aligned}$$

26b $$\begin{aligned}{} & {} \hspace{-2cm}c_2(t)=\frac{1}{2}\bigg (\mathcal {M}_+(t)+\mathcal {M}_-(t)\bigg )c_2(0) +\frac{1}{2}\bigg (\mathcal {M}_+(t)-\mathcal {M}_-(t)\bigg )c_1(0), \end{aligned}$$

where, the survival amplitudes $\mathcal {M}_{\pm }(t)=\mathcal {L}^{-1}\bigg (\frac{1}{s+F(s)\pm iD}\bigg )$ are given by

27 $$\begin{aligned} \mathcal {M}_{\pm }(t)=\sum _{i=1}^3\frac{ (q_{\pm }^i+u_+)(q_{\pm }^i+u_-)}{\prod _{j\ne i=1}^{3}(q_{\pm }^i-q_{\pm }^j)}e^{ q_{\pm }^i\lambda t}, \end{aligned}$$

with $q_{\pm }^i (i= 1, 2, 3)$ are roots of the following cubic equations

28 $$\begin{aligned} q_{\pm }^{3}+(y_1\mp y_2)q_{\pm }^2+ \left( u_+u_-+\frac{\gamma }{4\lambda }\mp y_1y_2\right) q_{\pm }+\frac{\gamma y_1}{8\lambda }\pm y_2u_+u_-=0, \end{aligned}$$

where $y_1=\frac{2\overline{\lambda }}{\lambda }$, $y_2=\frac{iD}{\lambda }$ and $u_{\pm }=\frac{y_1\pm \beta (y_1+2i\omega _o/\lambda)}{2}$. With substitution (26a) and (26b), respectively, into the reduced density matrices (15b) and (15a), and then using the $\Delta E_{A(B)}=\texttt {Tr}\{\rho _{A(B)}(t) H_{A(B)}\}-\texttt {Tr}\{\rho _{A(B)}(0) H_{A(B)}\},$ the internal energy of the charger and battery are deduced as

29 $$\begin{aligned} \Delta E_A=\omega _0\left( |c_1(t)|^2-|c_1(0)|^2\right) ,\quad \quad \Delta E_B=\omega _0\left( |c_2(t)|^2-|c_2(0)|^2\right) . \end{aligned}$$

We note that according to the above equations, energy that the charger loses at the end of charging process, i.e., $|\Delta E_A|$ and stored energy of battery $\Delta E_B$ satisfy the inequality $|\Delta E_A|\ge \Delta E_B$. This means that due to the interaction between the charger and cavity, some of its energy leaks into the cavity before being transferred to the battery. On the other hand, by substitution Eq. (15b) into (4), ergotropy of the battery reads (with $\mathcal {W}_{max}=\omega _0$)

30 $$\begin{aligned} \mathcal {W}=\mathcal {W}_{max}\left( 2|c_2(t)|^2-1\right) \Theta \left( |c_2(t)|^2-\frac{1}{2}\right) , \end{aligned}$$

where $\Theta (x-x_0)$ is the Heaviside function, which satisfies $\Theta (x-x_0)=0$ for $x<x_0$, $\Theta (x-x_0)=\frac{1}{2}$ for $x=x_0$ and $\Theta (x-x_0)=1$ for $x>x_0$.

**Figure 2:**
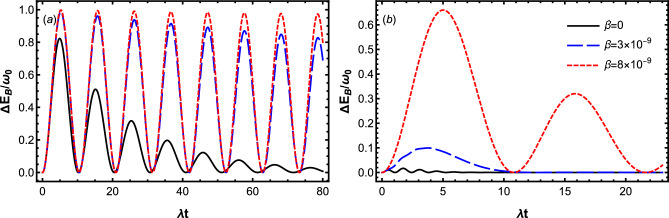
Dynamics of the stored energy $\Delta E_B$ for the different values of $\beta$ by setting $\omega _0=1.5\times 10^9\lambda$, $D=0.3\lambda$ and $\Delta =0$. The panels (**a**) displays the Markovian dynamics with $\gamma =0.1 \lambda$, while the panels (**b**) displays the non-Markovian dynamic with $\gamma =20\lambda$.

**Figure 3:**
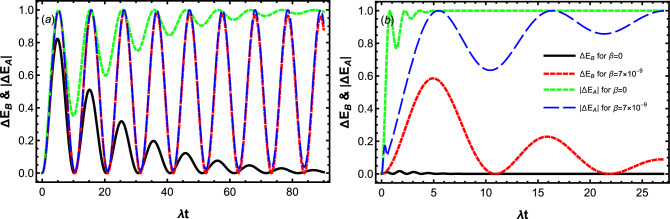
Dynamics of the stored energy $\Delta E_B$ and internal energy of charger $|\Delta E_A|$ for the different values of $\beta$ by setting $\omega _0=1.5\times 10^9\lambda$, $D=0.3\lambda$ and $\Delta =0$. The panels (**a**) displays the Markovian dynamics with $\gamma =0.1 \lambda$, while the panels (**b**) displays the non-Markovian dynamic with $\gamma =20\lambda$.

## Numerical results and discussion

In this section, we will analyze the charging dynamics of the introduced open moving-battery in the weak and strong coupling regimes. In particular, we explore the role of the movement of QB on the dynamical behavior of performance indicators including stored energy, ergotropy and efficiency. In our following analysis, we choose the optical regime parameters^64,65^ and set the qubit transition frequency as $\omega _0=1.5\times 10^{9}\lambda$. In what follows, we consider an initial condition in which the battery is initially empty and the charger has the maximum energy, i.e. $c_1(0)=1$, $c_2(0)=0$.

**Figure 4:**
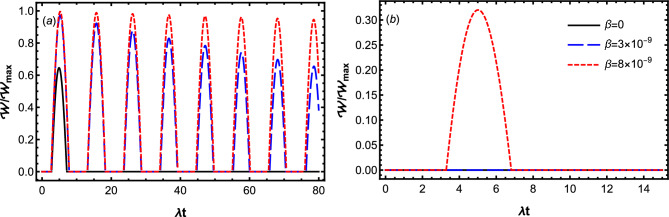
Dynamics of ergotropy $\mathcal {W}$ for the different values of $\beta$ by setting $\omega _0=1.5\times 10^9\lambda$, $D=0.3\lambda$ and $\Delta =0$. The panels (**a**) displays the Markovian dynamics with $\gamma =0.1 \lambda$, while the panels (**b**) displays the non-Markovian dynamic with $\gamma =20\lambda$.

In Fig. 2, we plot the Markovian and non-Markovian dynamics of the stored energy $\Delta E_B$ for the initial state $\left| \Psi (0)\right\rangle =\left| e\right\rangle _{A}\left| g\right\rangle _{B}\otimes \left| 0\right\rangle _{R_A}\left| 0\right\rangle _{R_B}$, by considering different values of the QB speed $\beta$. In panel (a), the battery is charged in the Markovian dynamics with $(\gamma =0.1\lambda )$, while in panel (b), it is charged in a non-Markovian dynamics with $(\gamma =20\lambda )$. Here we consider a situation at which the charger and battery’s qubits are both in resonance with the reservoir modes by setting $\Delta =0$. According to this figure, the positive impact of the translational motion of the charger and battery’s qubits in controlling the stored energy of battery is clearly visible in both Markovian and non-Markovian charging processes. As can be seen in both Fig. 2a and b, when the charger and battery’s qubits are at rest inside their cavity reservoirs, the stored energy in the battery $\Delta E_B$ decays into zero at sufficiently long times. However the rate of these decays decreases regularly by gradual growth of the qubit velocity, and therefore the energy stored in the battery and consequently the charging process is strongly protected from the environmental noises. Comparing Fig. 2a with b clearly reveals a fundamental difference between Markovian and non-Markovian charging processes. The maximal amount of stored energy in the Markovian charging process is more than that of the non-Markovian charging process. The reason stems from the nature of the qubit-cavity coupling. In the non-Markovian charging process, the coupling strength of charger’s qubit to the cavity modes is greater than its coupling to the battery’s qubit, therefore, the initial internal energy of charger has more tendency to evolve toward the reservoir than to the battery. Moreover, since the motional effect of QB has been included in battery-cavity and charger-cavity coupling strength, it seems that increasing speed of QB decreases the charger-cavity coupling strength in favor of the battery-charger coupling strength, which increases the stored energy of the battery.

To answer the question of why the stored energy exhibits oscillating-decay behavior, we give a concrete explanation as follows. We notice that in a closed QB the energy excitation remains in the battery-charger system; it is transferred from the charger to the battery and then comes back to the charger again. Therefore, the stored energy of the battery oscillates harmonically with the charging time. Damping of the storage energy occurs in the open QB, albeit only when the excitation in the battery-charger system escapes to the environment due to the system-environment interaction. In this case, the stored energy of battery damps monotonously under the Markovian dynamics, while it damps oscillatory under the non-Markovian dynamics due to the memory effects of the environment. However, the possibility of remaining the excitation in the battery-charger system results in an oscillating-decay dynamics of energy stored in the open QBs. Based on the above considerations, such a dynamical behavior can be observed in both the Markovian and non-Markovian regimes. In our charging protocol, because the initial state $\left| \Psi (0)\right\rangle =\left| e\right\rangle _{A}\left| g\right\rangle _{B}\otimes \left| 0\right\rangle _{R_A}\left| 0\right\rangle _{R_B}$ is invariant under the battery-reservoir interaction Hamiltonian $H_{B-R_B}$, the dynamics of the battery-charger system is mainly determined by $H_{A-B}+H_{A-R_A}$. While, $\left| \Psi (0)\right\rangle$ can be damped to $\left| g\right\rangle _{A}\left| g\right\rangle _{B}\otimes \left| 1\right\rangle _{R_A}\left| 0\right\rangle _{R_B}$ by $H_{A-R_A}$, it can be also transferred into $\left| g\right\rangle _{A}\left| e\right\rangle _{B}\otimes \left| 0\right\rangle _{R_A}\left| 0\right\rangle _{R_B}$ under the dipole–dipole interaction Hamiltonian $H_{A-B}$. Accordingly, the energy excitation of the initial state $\left| \Psi (0)\right\rangle$ can stay in the battery-charger system thanks to the dipole–dipole interaction, which leads to the oscillating-decay dynamics of the stored energy.

In order to get more insight to this area and a deeper understanding of the relationship between the charger and battery energy, in Fig. 3 we have illustrated the energy stored in the battery at the end of charging process as well as the energy that the charger loses at the same time. Here $\Delta E_B$ and $|\Delta E_A|$ have been plotted as a function of the dimensionless time $\lambda t$ for the qubit velocities $\beta =0$ and $\beta =7\times 10^{-9}$ in the Markovian and non-Markovian regimes. In the non-Markovian charging process, $|\Delta E_A|$ is much more than $\Delta E_B$ for a given $\beta$ as shown in Fig. 3b. This implies that the internal energy of the charger is not completely transferred to the battery. Figure 3b also shows that, when the charger and battery’s qubits are at rest inside their cavity reservoirs, the charger’s qubit immediately loses a large amount of its initial energy without being transferred to the battery. However, increasing the qubit velocity (decreasing the ratio of charger-cavity coupling strength to battery-charger coupling strength) during the non-Markovian process, decreases the initial loss-rate of the charger, and therefore improves the energy transfer in the charging processes.

The relationship between the charger and battery energy in the Markovian charging process is drastically different from that in the non-Markovian charging process. One can infer from Fig. 3a that, although for the static battery-charger system ($\beta =0$), the total energy of the charger can be transferred to the battery, $|\Delta E_A|=\Delta E_B$ satisfy just in the short charging Markovian process. Interestingly, when the qubits move with the velocity $\beta =7\times 10^{-9}$, $|\Delta E_A|=\Delta E_B$ holds at any charging time. So, we conclude again that a robust Markovian charging against the arisen dissipation can be achieved, when the qubits move with higher velocities.

**Figure 5:**
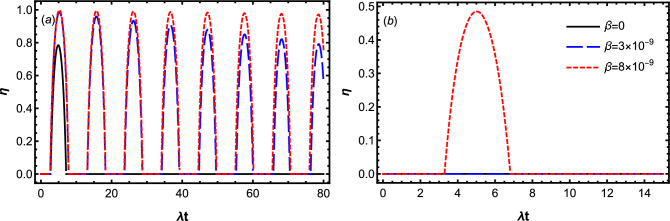
Dynamics of efficiency $\eta$ for the different values of $\beta$ by setting $\omega _0=1.5\times 10^9\lambda$, $D=0.3\lambda$ and $\Delta =0$. The panels (**a**) displays the Markovian dynamics with $\gamma =0.1 \lambda$, while the panels (**b**) displays the non-Markovian dynamic with $\gamma =20\lambda$.

In the following, we examine the influence of translational motion of the battery-charger system on the dynamics of ergotropy. In Fig. 4, we plot $\mathcal {W}/\mathcal {W}_{max}$ as a function of $\lambda t$ for the different values of $\beta$ in the Markovian (Fig. 4a) and non-Markovian (Fig. 4b) regimes. Our numerical results in Fig. 4a and b illustrate that, the effect of translational motion of QB on the ergotropy is also constructive in both Markovian and non-Markovian regimes. Figure 4b shows that, in the non-Markovian regime, in the cases of stationary ($\beta =0$) and slowly moving ($\beta =3\times 10^{-9}$) qubits, we are not able to extract useful work from the QB, but in this regime a considerable work can be extracted, as the qubits move with a higher velocity ($\beta =8\times 10^{-9}$). Our numerical results in Fig. 4a illustrate that, the effect of translational motion of QB on the ergotropy is more considerable in the Markovian case. We observe that, in the Markovian regime, increasing the speed of QB (decreasing the qubit-reservoir coupling) not only boosts the ergotropy, but also increases the number of time zones in which work can be extracted. Accordingly, a strong robust charging process can be established in the higher speed limit, in which the extractable work approaches to its maximum value.

In this stage, we examine the effect of translational motion of QB on the Markovian and non-Markovian charging efficiency. The results for Markovian and non-Markovian charging processes are presented in Fig. 5a and b, respectively. Here we consider the same parameter values as in Fig. 4. Comparing Figs. 4 and 5 reveals that both ergotropy and efficiency are positively affected by the translational motion of QB. However the efficiency is influenced more than the ergotropy; the amount of increment in efficiency is more than the ergotropy in both Markovian and non-Markovian charging processes.

**Figure 6:**
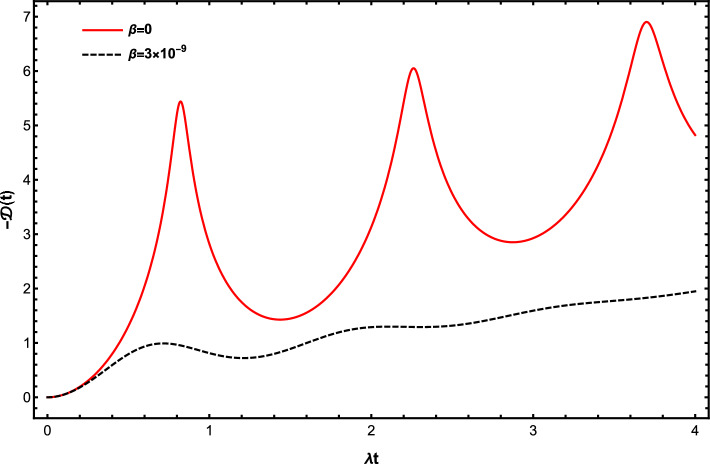
Dynamics of decoherence function for the static and moving battery-charger system. Here we set $\omega _0=1.5\times 10^9\lambda$, $D=0.3\lambda$, $\Delta =0$ and $\gamma =10\lambda$.

Finally, we investigate the impact of qubits motion on the quantum decoherence of the battery-charger system. It is worth to remark that decreasing the loss of quantum coherence (decreasing decoherence rate) between the battery and charger is a valuable step toward enhancing the stored energy of the open QBs. To identify decoherence we define the decoherence function by exploiting the off-diagonal elements of the battery-charger density matrix $\rho _{AB}(t)=\textrm{Tr}_{R_A,R_B}\{\left| \Psi (t)\right\rangle \langle \Psi (t)|\}$ in the following form^63^

31 $$\begin{aligned} \mathcal {D}(t)=\textrm{ln}\bigg |\frac{c_1(t)c_2^{*}(t)}{c_1(0)c_2^{*}(0)}\bigg |. \end{aligned}$$

In Fig. 6, we illustrate dynamics of decoherence function $\mathcal {D}(t)$ for $\beta =0$ and $\beta =3\times 10^{-9}$. Here we choose the initial conditions $c_1(0)=c_2(0)=\frac{1}{\sqrt{2}}$ and set $\gamma =10\lambda$. The plots displayed in this figure give evidence that interesting results can be obtained by increasing the velocity of the charger and battery’s qubits. In despite of decreasing the oscillating nature (associated to the degree of non-Markovianity), the initial quantum coherence is strongly protected against the environmental induced dissipation, which leads to enhance the performance of the QB.

## Outlook and summary

To summarize, we proposed a mechanism for robust charging process of an open qubit-based quantum battery (QB) whose robustness can be well controlled by the translational motion of the charger and battery in both Markovian and non-Markovian dynamical regimes. Both the battery and charger’s qubits move with a same velocity inside two separated identical environments, and are directly coupled by the dipole–dipole interaction. We showed that the stored energy, ergotropy and efficiency of the moving QB regularly increased with the gradual growth of the speed of charger and battery, thereby improving its charging performance due to a corresponding decrease of the decoherence rate as shown in Fig. 6. To gain a physical perspective on the constructive role of the translational motion of QB in controlling the charging process, we note that the impact of qubit velocity on the charging performance arises from the attachment of qubits velocity to the qubit-reservoir coupling strength [see Eq. (8)]. Although the sine functionality of the shape function $f_k(vt)$ makes it impossible to establish a linear relationship between the qubits velocity and strength of the qubit-reservoir coupling, what is certain is that the motion of the the charger and battery’s qubits gives rise to weakening the strength of the qubit-reservoir coupling. Due to the fact that the QB is charged with the help of the dipole–dipole interaction, a weak qubit-reservoir coupling is sufficient to maintain the initial coherence of the battery-charger system and consequently to create a robust charging process.

Our results represent a novel control strategy to have a robust QB with a natural implementation in cavity-QED context. The strategy can be easily implemented also in the circuit-QED setups where the qubit position slowly varies linearly with time and also the qubit-cavity interaction is tuned through a sinusoidal position-dependent coupling^66^.

In perspective, we believe that this strategy can be used to control the performance of the discharging of a qubit-based QB to an available consumption hub. Further efforts in this field can be devoted to use the proposed strategy for improving the performance of the two-photon based charging process where the moving-QB is coupled with a cavity reservoir by means of a two-photon relaxation.

## Data Availability

The datasets used and analysed during the current study available from the corresponding author on reasonable request.
